# Desirable Difficulties in Spatial Learning: Testing Enhances Subsequent Learning of Spatial Information

**DOI:** 10.3389/fpsyg.2018.01701

**Published:** 2018-09-11

**Authors:** Jonathan Bufe, Alp Aslan

**Affiliations:** Department of Psychology, Martin Luther University of Halle-Wittenberg, Halle, Germany

**Keywords:** desirable difficulties, test-enhanced learning, forward testing effect, spatial memory, proactive interference

## Abstract

Previous work has shown that testing can enhance learning and retention of subsequently studied new information. The present study investigated this *forward testing effect* in spatial memory. In two experiments, participants studied four successively presented 3 × 3 arrays, each composed of the same nine objects. They were asked to memorize the locations of the objects which differed across the four arrays. Following presentation of Arrays 1–3, memory for the object locations of the respective array was tested (testing condition), or the array was re-presented for additional study (restudy condition). Thereafter, Array 4 was presented and tested in both the testing and the restudy condition. In Experiment 1, testing was self-paced, whereas in Experiment 2, testing time was controlled by the experimenter. Consistent across the two experiments, testing was found to enhance location memory for Array 4, relative to restudying. Furthermore, testing also reduced the number of confusion errors (i.e., the tendency to misplace objects to locations on which they had appeared previously) made during recall of Array 4, suggesting that testing reduced the interference potential of prior information. The results indicate that testing can enhance subsequent learning of spatial information by reducing the build-up of proactive interference from previously studied information.

## Introduction

An intriguing implication from recent research is that introducing difficulties into the learning process can affect learning and memory in desirable ways (Schmidt and Bjork, 1992; Bjork, 1994). A prominent example of such a *desirable difficulty* that has attracted the attention of researchers and educators alike is testing. Indeed, numerous studies have found that taking a test on previously studied information can lead to superior long-term retention of that information, compared to other, supposedly less effortful learning “activities” such as doing nothing or restudying the information. This beneficial effect of testing, hereinafter called the *backward testing effect* (BTE; Pastötter and Bäuml, 2014), has been demonstrated using a wide range of learning materials and employing both laboratory and classroom settings (e.g., Hogan and Kintsch, 1971; Wheeler and Roediger, 1992; Roediger and Karpicke, 2006; Carpenter and Pashler, 2007; McDaniel et al., 2007; Karpicke and Roediger, 2008; for a review, see Roediger and Butler, 2011).

The focus of the current research was another, less well-known beneficial effect of testing, called the *forward testing effect* (FTE; Tulving and Watkins, 1974; Pastötter and Bäuml, 2014). The FTE has typically been demonstrated in studies in which participants were required to learn several sets of information in succession. Such elongated study sessions are known to foster the build-up of proactive interference, which is the deleterious effect of prior learning on retention of subsequently studied target information (Underwood, 1957; Crowder, 1976; Anderson and Neely, 1996). Szpunar et al. (2008), for instance, asked three groups of participants to study five lists of words in anticipation of a final cumulative recall test. The three participant groups differed in the activity that followed presentation of each of the first four lists, with one group doing an irrelevant filler task after Lists 1–4, one group restudying Lists 1–4, and one group being immediately tested on Lists 1–4. Szpunar et al. (2008) found that immediate testing of Lists 1–4 improved memory for the subsequently studied List 5, with the tested group recalling about twice as many List 5 words as the other two groups. Moreover, immediate testing of Lists 1–4 also decreased the number of intrusions from these lists produced during recall of List 5, compared to the other two conditions, suggesting that testing can counteract the build-up of proactive interference. In particular, the findings indicate that, in addition to enhancing memory for previously studied (and tested) information (BTE), testing can also enhance learning and retention of subsequently studied new information (FTE).

More recent work has conceptually replicated and extended Szpunar et al.’s (2008) original findings. Weinstein et al. (2011), for instance, let participants study four lists of face-name pairs. Following study of each of the first three lists, participants engaged in an irrelevant distractor task or received an immediate test on the names given the associated faces as retrieval cues. Results revealed that tested participants showed higher recall of the fourth list, and fewer prior-list intrusions during List 4 recall than non-tested participants. Analogous results were reported using vocabulary pairs (Cho et al., 2016), object-name pairs (Pastötter et al., 2013), and expository text passages (Wissman et al., 2011). In a recent study, Szpunar et al. (2013) let participants watch an introductory statistics video divided into four 5-min segments. Mimicking the typical pattern of results, participants who were tested after each of the first three segments performed better on a test assessing the contents of Segment 4 than participants who restudied the first three segments. Together, these findings evince that the FTE is a fairly robust and general phenomenon that can be obtained across a variety of experimental setups and learning contents (for a recent review, see Yang et al., 2018).

Several explanations of the FTE have been proposed, emphasizing either improved encoding or improved retrieval as the source of the effect. Retrieval-based explanations assume that testing promotes list segregation, which reduces the size of the mental search set and thus allows for more focused memory search when it comes to recall of the target list (Szpunar et al., 2008; Bäuml and Kliegl, 2013). Encoding-based explanations, on the other hand, assume that testing already enhances the encoding of subsequently studied information. According to these explanations, testing induces a reset of encoding processes, making the encoding of subsequently studied information as effective as the previously studied information (Pastötter et al., 2011, 2018), or leads to a strategy shift such that the later presented information is studied using more efficient encoding strategies than the earlier presented information (Wissman et al., 2011; Chan et al., 2018). Of course, these different explanations of the FTE need not be mutually exclusive, and it is possible that both encoding and retrieval processes are involved when testing insulates against the build-up of proactive interference (for a comprehensive overview of theoretical accounts of the FTE, see Yang et al., 2018).

Research on proactive interference has traditionally focused on verbal (content) information, and so has research on the reducibility of proactive interference through interpolated testing. In fact, only few studies have examined the FTE using nonverbal information (e.g., Lee and Ahn, 2018; Yang and Shanks, 2018), and, in particular, no study has yet examined whether the effect generalizes to spatial information. This is surprising given that proactive interference can build-up not only in verbal memory, but also in spatial memory. For instance, in daily life, we often find ourselves in search of our keys, mobile phones, or cars in a parking area, being momentarily unable to remember the particular location where we had placed the sought-after object last. At least part of our problem may arise from the fact that earlier locations of the object interfere with the current one, making the memory search for the target location more difficult, if not entirely unsuccessful.

Importantly, there is not only anecdotal, but also empirical evidence for proactive interference in spatial memory, although there is relatively little research that examined the issue in the long-term memory domain. In one of the few studies, Elmes (1988) employed a concentration game in which participants learned the positions of cards arranged in an array. The cards were provided face down and participants were asked to successively turn over two cards at a time. If the pictures of the two cards matched, they remained face up; if the pictures mismatched, they were turned over again. This procedure was continued until all cards were uncovered. Then, the cards were turned face down again, and after a retention interval, participants’ memory for the card positions was assessed. The manipulation of interest was the number of (non-target) arrays studied prior to the target array. Elmes found in two experiments that location memory for the target array decreased as the number of previously provided (non-target) arrays increased, thus confirming the subjective impression that proactive interference can be a significant source of forgetting in spatial memory (see also Smith et al., 1995; Postma et al., 2018).

The finding of proactive interference in spatial memory raises the question of whether such interference can be reduced by interpolated testing. Previous work has provided evidence that testing can affect spatial memory, although this evidence comes exclusively from studies on the BTE. Carpenter and Pashler (2007), for instance, let participants learn two maps, one through two successive study phases, the other through one study phase followed by a test phase in which missing features from the map together with their locations had to be covertly recalled. When later asked to draw the two maps, participants’ drawings were more accurate in the study-test than in the study-study condition, suggesting that testing can enhance retention of previously studied spatial information (for similar findings, see Rohrer et al., 2010; Carpenter and Kelly, 2012; Kelly et al., 2015). Concluding from these findings that testing can also reduce the build-up of proactive interference in spatial memory, and in this way, enhance learning and retention of subsequently studied new spatial information would be premature, however. Indeed, there is evidence–though from separate studies–that the BTE and the FTE follow different developmental trajectories, with the former developing earlier in life than the latter (e.g., Fritz et al., 2007; Aslan and Bäuml, 2015), suggesting that different mechanisms operate in these two types of testing effects.

The goal of the present study was to conceptually replicate and extend on previous research by (i) confirming the finding of proactive interference in spatial memory (e.g., Elmes, 1988), and (ii) examining for the first time whether such proactive interference in spatial memory can be counteracted by interpolated testing. In two experiments, participants studied four successively presented 3 × 3 arrays composed of the same nine objects, but each time in a different spatial arrangement. Following presentation of each of the first three (non-target) arrays, memory for the object locations of the respective array was tested (testing condition), or the array was re-presented for additional study (restudy condition). The critical variable was participants’ location memory performance on the fourth (target) array which was tested in both the testing and the restudy condition. In Experiment 1, testing was self-paced, whereas in Experiment 2, testing time was controlled by the experimenter. In both experiments, we expected to replicate the finding of proactive interference in spatial memory (e.g., Elmes, 1988) by obtaining poorer location memory for the fourth array in the restudy condition than for the first array in the testing condition (which served as an interference-free baseline). More important, if testing reduces the build-up of proactive interference and enhances learning and retention of subsequently studied spatial information, then we should find location memory for the fourth array to be higher in the testing condition than in the restudy condition.

## Experiment 1

### Methods

#### Participants

*A priori* power analysis using G^∗^Power 3 (Faul et al., 2007) with a significance level of α = 0.05, a (conservatively estimated) medium effect size of *d* = 0.40, and a desired power of (1-β) = 0.75 revealed a sample size of *N* = 46. Based on this analysis, we examined 48 adults (*M* = 25.6, *SD* = 4.7 years; 26 females), which were recruited from both the community and the Martin Luther University of Halle-Wittenberg, Germany. All participants gave written informed consent and were tested individually. Students of Martin Luther University of Halle-Wittenberg received course credit for their participation. The experiment was carried out in accordance with the recommendations of the Ethics Committee of the Faculty of Medicine at Martin Luther University of Halle-Wittenberg, Germany. An ethical approval was not required for this experiment as per the committee’s guidelines and national regulations.

#### Materials

**FIGURE 1:**
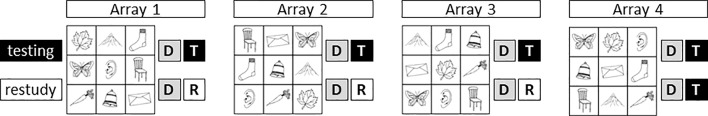
Overview of the experimental procedure: Participants studied four successively presented 3 × 3 arrays composed of the same nine objects, but each time in a different spatial arrangement. Following presentation of Arrays 1–3 and a distractor task (D), memory for the object locations of the respective array was tested (T: testing condition), or the array was re-presented for additional study (R: restudy condition). Array 4 was followed by a distractor task and was tested in both the testing and the restudy condition.

The study materials consisted of 18 black-and-white pictures of easily identifiable objects drawn from the Snodgrass and Vanderwart (1980) norms. The 18 pictures were randomly divided into two sets of nine pictures each (set A and set B). The nine pictures of each set were printed on sheets of paper sized 16.5 × 16.5 cm. For each set, four different 3 × 3 arrays were (pseudo-randomly) constructed such that, across the four arrays, no object appeared on the same position twice (see **Figure 1**). These arrays were used in study/restudy trials. Moreover, we also prepared sheets of paper with empty 3 × 3 arrays as well as nine 5.1 × 5.1 cm picture cards of the objects for use in test trials.

#### Design

The experiment had a 2 × 4 design with the within-subjects factors of *Learning Condition* (testing and restudy) and *Array Position* (Arrays 1–4). In both learning conditions, participants studied four consecutively presented 3 × 3 arrays. The arrays were composed of the same nine objects in different spatial arrangements. The two learning conditions differed in whether, following presentation of each of the first three arrays, the arrays were tested (testing condition) or were re-presented for additional study (restudy condition).

#### Procedure

The experimental procedure is shown in **Figure 1**. Participants were initially informed that they would be presented with four 3 × 3 arrays containing the same nine objects in different spatial arrangements, and that they should memorize the locations of the objects within each arrangement in anticipation of an upcoming memory test. Participants were further told that, if an array was tested immediately after its presentation, they should still continue keeping it in mind, as all arrays would be tested on a delayed final test. Following the initial instruction, participants were successively provided with the four arrays of one set of objects (set A or set B), and were asked to memorize the spatial arrangement of the arrays. Each of the four arrays was presented for 30 s and was immediately followed by a 30-s distractor task in which participants counted backward in steps of threes. The procedure for the two learning conditions (testing and restudy) differed in the activity that followed the backward-counting task after Arrays 1–3: In the restudy condition, each array was presented again for another 30 s, and participants were asked to restudy the spatial arrangement of the array; in the testing condition, participants were provided with empty 3 × 3 arrays, and were asked to recall the spatial arrangement of the just-presented array and reconstruct it using the picture cards of the nine objects. The test was self-paced. The procedure for Array 4 differed from the other three arrays in that Array 4 was tested after the 30-s backward-counting task in both the testing and the restudy condition. Following another 1-min backward-counting task, participants were given the announced final test for all four arrays but the results are not reported here (see **Supplementary Materials**, for further information). After a short break, the second learning condition (restudy or testing) was administered using the other set of objects (set B or set A). The order of the two learning conditions (testing first or restudy first) was counterbalanced across participants, as was the assignment of the two object sets (set A, set B) to the two learning conditions and the four array positions.

### Results

The raw data of the two experiments have been made publicly available via the Open Science Framework (OSF) at http://osf.io/x97zp/.

**FIGURE 2:**
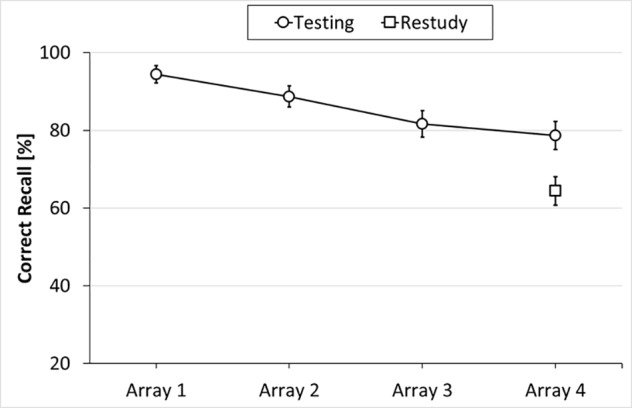
Results of Experiment 1: Mean percentage of correctly recalled object locations as a function of Learning Condition (testing, restudy) and Array Position (Arrays 1–4). Error bars represent standard errors.

The results of Experiment 1 are shown in **Figure 2**. As can be seen from the figure, object location memory was poorer for Array 4 in the restudy condition than for (the interference-free) Array 1 in the testing condition [64.4% vs. 94.4%, *t*(47) = 7.69, *SE* = 0.04, *p* < 0.001, *d* = 1.16], reflecting substantial proactive interference in the restudy condition. As can further be seen, performance within the testing condition varied across the four arrays [Array 1: 94.4%, Array 2: 88.7%, Array 3: 81.7%, and Array 4: 78.7%; *F*(3,141) = 7.86, *p* < 0.001, η^2^ = 0.14], suggesting that there was also significant build-up of proactive interference in the testing condition. Specifically, *post hoc* tests (Holm-corrected, one-tailed) revealed significant differences between Arrays 1 and 3 (*p* < 0.001), Arrays 1 and 4 (*p* < 0.001), and Arrays 2 and 4 (*p* = 0.038). The other pairwise comparisons did not reach significance. Importantly, location memory for Array 4 was higher in the testing condition (78.7%) than in the restudy condition (64.4%), indicating that testing significantly reduced the build-up of proactive interference [*t*(47) = 3.10, *SE* = 0.05, *p* = 0.003, *d* = 0.45].

We also examined confusion errors, i.e., the participants’ tendency to misplace objects to locations on which they had appeared previously. Such confusion errors reflect the lingering (negative) effects of prior learning, and thus can be regarded another index of proactive interference. Results revealed that, in the testing condition, confusion errors increased from Arrays 2–4 [0.9% vs. 9.0%; *t*(47) = 4.28, *SE* = 0.02; *p* < 0.001, *d* = 0.62], with confusion errors in Array 3 (7.2%) falling in-between, confirming that there was significant build-up of proactive interference. Importantly, confusion errors in Array 4 were lower in the testing condition than in the restudy condition [9.0% vs. 16.0%, *t*(47) = 2.67, *SE* = 0.03, *p* = 0.010, *d* = 0.39], indicating that testing of Arrays 1–3 reduced these arrays’ potential to interfere with recall of Array 4, compared to restudy of Arrays 1–3.

## Experiment 2

In Experiment 1, the time participants were given for restudy of Arrays 1–3 was determined by the experimenter (30 s), whereas testing of the arrays was self-paced. While this was done deliberately to avoid time pressure, one could argue that the procedural difference between the two learning conditions might have influenced the overall pattern of results. To address this potential objection, we conducted a second experiment in which we matched the processing time between the testing and the restudy condition.

### Methods

#### Participants

*A priori* power analysis using G^∗^Power 3 (Faul et al., 2007) with a significance level of α = 0.05, an effect size of *d* = 0.45 (estimated from Experiment 1), and a desired power of (1-β) = 0.75 revealed a sample size of *N* = 37. Based on this analysis, we examined forty adults (*M* = 22.4, *SD* = 2.3 years; 23 females), which were recruited from both the community and the Martin Luther University of Halle-Wittenberg, Germany. All participants gave written informed consent and were tested individually. Students of Martin Luther University of Halle-Wittenberg received course credit for their participation. The experiment was carried out in accordance with the recommendations of the Ethics Committee of the Faculty of Medicine at Martin Luther University of Halle-Wittenberg, Germany. An ethical approval was not required for this experiment as per the committee’s guidelines and national regulations.

#### Materials, Design, and Procedure

Materials, design, and procedure were identical to Experiment 1, except for the following three changes: First, we replaced one object (*mountain*) that was not always correctly identified in Experiment 1 by a new object (*star*). Second, we constructed two new, randomly composed sets of nine objects from the 18 items used. Third, and most importantly, we matched the procedure between the two learning conditions by giving participants 30 s in both restudy and test trials.

### Results

**FIGURE 3:**
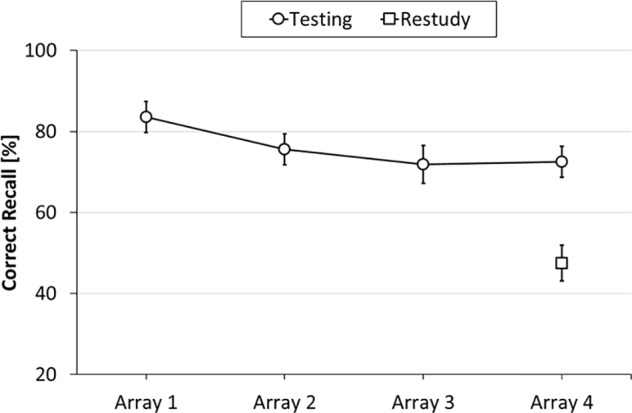
Results of Experiment 2: Mean percentage of correctly recalled object locations as a function of Learning Condition (testing, restudy) and Array Position (Arrays 1–4). Error bars represent standard errors.

The results of Experiment 2 are shown in **Figure 3**. As can be seen from the figure, object location memory was poorer for Array 4 in the restudy condition than for (the interference-free) Array 1 in the testing condition [83.6% vs. 47.5%, *t*(39) = 7.94, *SE* = 0.05, *p* < 0.001, *d* = 1.26], reflecting substantial proactive interference in the restudy condition. As can further be seen, performance within the testing condition varied across the four arrays [Array 1: 83.6%, Array 2: 75.6%, Array 3: 71.9%, and Array 4: 72.5%; *F*(3,117) = 2.99, *p* = 0.034, η^2^ = 0.07], suggesting that there was also significant build-up of proactive interference in the testing condition. Specifically, *post hoc* tests (Holm-corrected, one-tailed) revealed significant differences between Arrays 1 and 3 (*p* = 0.048), and Arrays 1 and 4 (*p* = 0.030). The other pairwise comparisons did not reach significance. Importantly, location memory for Array 4 was higher in the testing condition (72.5%) than in the restudy condition (47.5%), indicating that testing significantly reduced the build-up of proactive interference [*t*(39) = 4.66, *SE* = 0.05, *p* < 0.001, *d* = 0.74].

Additional analyses revealed that, in the testing condition, confusion errors increased from Arrays 2–4 [3.3% vs. 7.5%; *t*(39) = 2.11, *SE* = 0.02; *p* < 0.042, *d* = 0.33], with confusion errors in Array 3 (6.1%) falling in-between, confirming that there was significant build-up of proactive interference. Importantly, confusion errors in Array 4 were lower in the testing condition than in the restudy condition [7.5% vs. 24.2%, *t*(39) = 4.60, *SE* = .04, *p* < 0.001, *d* = 0.73], indicating that testing of Arrays 1–3 reduced these arrays’ potential to interfere with recall of Array 4, compared to restudy of Arrays 1–3.

## Discussion

Prior research has shown that testing can enhance subsequent learning of verbal information (e.g., Szpunar et al., 2008). The present study was the first to examine this FTE in spatial memory. Consistent across two experiments, we found that testing the locations of objects on three previously studied arrays improved location memory for the same objects on a subsequently studied fourth array. Also consistent across the two experiments, testing reduced the number of confusion errors made during recall of the fourth array, i.e., tested participants showed less tendency to erroneously misplace objects to locations on which they had appeared previously. These findings indicate that testing can enhance subsequent learning of spatial information by counteracting the build-up of proactive interference from previously studied information.

A noteworthy feature in the present data is that, in both experiments, testing reduced, but did not eliminate proactive interference. Indeed, although testing of Arrays 1–3 enhanced location memory for Array 4, relative to restudying, performance within the testing condition decreased from (the interference-free) Arrays 1–4, suggesting that there was still build-up of proactive interference across the four arrays. This result is well in line with prior research that, using similar study procedures with intervening tests, reported significant proactive interference in spatial memory (Elmes, 1988; Smith et al., 1995; Postma et al., 2018). Yet, the result contrasts with previous studies that, examining the FTE with verbal materials, often did not find significant build-up of proactive interference across lists in the testing condition (e.g., Szpunar et al., 2008; Pastötter et al., 2011; Aslan and Bäuml, 2015). However, these previous studies employed relatively small sample sizes (only 12–18 participants per condition), raising the possibility that a present proactive-interference effect may have gone undetected. Obviously, more research is needed to determine whether the suggested dissociation in the effects of testing between verbal and spatial memory arose from specifics of the particular experimental setups, or reflects a more fundamental difference between the two types of memory.

Theoretical accounts of the FTE have emphasized either retrieval or encoding processes to explain the effect in verbal memory. For instance, it has been argued that testing enhances the segregation of target and non-target information, which reduces the size of the functional memory search set, and thus leads to more focused memory search during attempts to retrieve the target information (Szpunar et al., 2008). Others have argued that testing may already enhance the encoding of subsequent information, either by inducing a reset of encoding processes (Pastötter et al., 2011), or by inducing a shift to a superior encoding strategy (Wissman et al., 2011). The present study was not designed to distinguish between these alternative explanations, and it is quite conceivable that both encoding and retrieval processes contributed to the FTE observed in our experiments. Still, the present results impose some important restrictions on the proposed theoretical accounts. For instance, the strategy-shift hypothesis would predict an increase, rather than a decrease, in memory performance from Arrays 1–4, a prediction that is challenged by the data. Furthermore, the above-reported finding that testing reduced but did not completely eliminate proactive interference suggests that the supposed segregation of target and non-target information and/or the supposed reset of encoding processes are less perfect in spatial memory than in verbal memory.

The current findings bear important implications for educational contexts where students often have to learn large amounts of information in succession. While much of this learning involves verbal (content) information, curricula in different disciplines may also require the learning of spatial information, including the learning of maps in geography, the learning of brain areas in neuroanatomy, or the learning of the spatial structure of molecules in chemistry. The present finding that the FTE is not restricted to verbal information but generalizes to spatial information thus is promising news for educators working in such disciplines. In particular, the finding indicates that testing represents a desirable difficulty by showing that testing can enhance subsequent learning of spatial information by insulating against the build-up of proactive interference.

## Ethics Statement

This study was carried out in accordance with the recommendations of the Ethics Committee of the Faculty of Medicine at Martin Luther University of Halle-Wittenberg, Germany. An ethical approval was not required for this experiment as per the committee’s guidelines and national regulations. All participants gave written informed consent.

## Author Contributions

AA developed the study concept and critically revised the manuscript. JB drafted the manuscript. Both authors contributed to the study design, analyzed and interpreted the data, and approved the final version of the manuscript for submission.

## Conflict of Interest Statement

The authors declare that the research was conducted in the absence of any commercial or financial relationships that could be construed as a potential conflict of interest.
