# Supplementary material for: Desirable Difficulties in Spatial Learning: Testing Enhances Subsequent Learning of Spatial Information
Source: Front Psychol. 2018 Sep 11;9:1701. doi: 10.3389/fpsyg.2018.01701 (PMC6141732; doi:10.3389/fpsyg.2018.01701)
Supplement: Supplementary file 1 [file Table_1.DOCX]

Supplementary Materials

Desirable Difficulties in Spatial Learning:
Testing Enhances Subsequent Learning of Spatial Information

Jonathan Bufe & Alp Aslan*

*** Corresponding author** (alp.aslan@psych.uni-halle.de)

# Procedures and Results of the Final Tests

# Experiment 1

*Procedure.* Participants in both learning conditions were asked to successively recall the previously studied arrays. Testing order was Array 1 🡪 Array 2 🡪 Array 3 🡪 Array 4. To reduce task demands, we provided participants with six of the nine object locations of each array as a retrieval cue, and asked them to merely recall the array’s remaining three object locations. Testing was self-paced.

*Results.* The results are shown in Table 1. A 2 x 4 analysis of variance (ANOVA) with the within-subjects factors of *Learning* *Condition* (testing, restudy) and *Array* *Position* (Array 1 to Array 4) revealed no significant effects (all *F*s < 1).

# Experiment 2

*Procedure.* The procedure in Experiment 2 was the same as in Experiment 1 except that Array 4 was tested first, and the remaining three arrays were tested thereafter in random order.

*Results.* The results are shown in Table 1. A 2 x 4 ANOVA with the within-subject factors of *Learning* *Condition* (testing, restudy) and *Array* *Position* (Array 1 to Array 4) revealed a significant main effect of *Array* *Position*, *F*(3,117) = 3.50, *p* = .018, *η^2^* = .04, reflecting higher recall in Array 4 than in the three other arrays (all *p*s < .05). There was also a significant interaction between the two factors, *F*(3,117) = 2.78, *p* = .044, *η^2^* = .02, reflecting the fact that testing improved object location memory for Array 4 (*p* = .019), but not for the remaining three Arrays (all *p*s > .195), relative to restudy.

**Table 1.** Mean proportion of correctly recalled object locations in the final tests of Experiment 1 and 2 as a function of *Learning* *Condition* (testing, restudy) and *Array* *Position* (Array 1 to Array 4).

|  |  | Array 1 | Array 2 | Array 3 | Array 4 |
| --- | --- | --- | --- | --- | --- |
| **Exp. 1** | testing | 0.71 | 0.70 | 0.80 | 0.81 |
|  | restudy | 0.83 | 0.77 | 0.77 | 0.81 |
| **Exp. 2** | testing | 0.62 | 0.68 | 0.69 | 0.91 |
|  | restudy | 0.73 | 0.63 | 0.63 | 0.72 |
